# Machine learning-based models for preoperative prediction of pituitary adenoma consistency: a systematic review and meta-analysis

**DOI:** 10.1007/s00701-026-06775-w

**Published:** 2026-01-24

**Authors:** Bardia Hajikarimloo, Ibrahim Mohammadzadeh, Salem M. Tos, Ali Mortezaei, Mohammad Amin Habibi

**Affiliations:** 1 Department of Neurological Surgery, University of Virginia, Charlottesville, VA USA; 2 Skull Base Research Center, Loghman-Hakim Hospital, Shahid Beheshti University of Medical Sciences, Tehran, Iran; 3 Student Research Committee, Gonabad University of Medical Sciences, Gonabad, Iran; 4 Department of Neurosurgery, Shariati Hospital, Tehran University of Medical Sciences, Tehran, Iran

**Keywords:** Pituitary adenoma, Tumor consistency, Radiomics, Machine learning, MRI, Preoperative prediction

## Abstract

**Background/objectives:**

The consistency of pituitary adenoma (PA) significantly impacts surgical difficulty and the extent of resection. Machine learning (ML) and radiomics have emerged as quantitative tools to predict tumor firmness from MRI-derived features. This systematic review and meta-analysis aimed to synthesize the diagnostic performance of ML-based models for preoperative prediction of PA consistency.

**Methods:**

PubMed, Embase, Scopus, and Web of Science were searched through September 2025. Studies developing or validating ML or deep learning (DL) models for predicting PA consistency were included. Pooled estimates of area under the curve (AUC), accuracy (ACC), sensitivity (SEN), specificity (SPE), and diagnostic odds ratio (DOR) were calculated with 95% confidence intervals (CIs).

**Results:**

Nine studies with 1,621 patients were analyzed. Algorithms included Extra Trees (ET), Random Forest (RF), Support Vector Machine (SVM), k-Nearest Neighbors (kNN), Logistic Regression (LR), Artificial Neural Network (ANN), and hybrid DL architectures. The pooled AUC was 0.92 (95% CI: 0.86–0.98), ACC 0.86 (95% CI: 0.79–0.92), SEN 0.80 (95% CI: 0.71–0.87), SPE 0.85 (95% CI: 0.80–0.89), and DOR 19.27 (95% CI: 10.27–36.17). Leave-one-out analyses confirmed robustness, and Egger’s tests indicated no significant publication bias.

**Conclusion:**

ML-based models demonstrate excellent pooled diagnostic accuracy in predicting PA consistency preoperatively, underscoring their value for individualized surgical planning. Future multicenter studies with standardized imaging and external validation are needed to optimize clinical translation.

**Supplementary Information:**

The online version contains supplementary material available at 10.1007/s00701-026-06775-w.

## Introduction

Pituitary adenomas (PAs) are the third most common intracranial tumors, making up about 10–20% of all primary brain tumors, with an estimated prevalence of 80–100 cases per 100,000 people [23, 37]. They typically develop from adenohypophyseal cells and encompass both functional and non-functional subtypes [28, 38]. Although histologically benign, their clinical significance arises from mass effects, compression of visual pathways, and hormone hypersecretion or deficiencies [9, 22, 44]. Advances in transsphenoidal endoscopic surgery have established resection as the primary treatment for symptomatic or enlarging tumors [19]. However, tumor consistency, ranging from soft and easily aspirated to firm and fibrous, remains a key factor during surgery, influencing the difficulty, extent of removal, and outcome [1, 20, 34]. Hard or fibrous adenomas often require sharp dissection, more extensive approaches, or ultrasonic aspirators, and are associated with higher rates of residual tumor and recurrence. Preoperative knowledge of tumor consistency can substantially improve surgical planning, risk anticipation, and patient counseling, particularly in cases where firm tumors may require extended dissection or alternative instrumentation [1, 20, 34].

Recent advances in artificial intelligence (AI) have introduced machine learning (ML) and radiomics as transformative tools in neuroimaging analysis [16–18]. Radiomics extracts quantitative, high-dimensional features from standard magnetic resonance imaging (MRI) sequences, capturing subtle aspects of texture, intensity, and spatial heterogeneity that are often imperceptible to the human eye [33]. When integrated with ML algorithms, these features can model complex relationships between imaging patterns and biological properties, providing noninvasive insights into tumor behavior [30, 35]. In the context of PAs, ML approaches have been increasingly studied as potential methods for predicting tumor consistency preoperatively, offering objective and reproducible alternatives to traditional qualitative assessments based on T1- or T2-weighted signal characteristics [6, 8, 11, 24, 27, 32, 39, 40, 45].

Despite promising initial results, the current literature remains varied, with significant differences in imaging protocols, segmentation methods, feature extraction pipelines, and intraoperative reference standards. Many studies are single-center and retrospective, which limits their generalizability and external validation. Additionally, the biological basis connecting radiomic signatures to histopathologic features, such as collagen density, fibrosis, or vascularity, remains only partially understood. To overcome these limitations, this systematic review and meta-analysis consolidates available evidence on ML-based models for preoperative prediction of PA consistency, aiming to determine pooled diagnostic performance, identify methodological weaknesses, and guide future clinical integration of artificial intelligence in pituitary surgery.

## Materials and methods

### Objective

The objective of this study was to systematically evaluate and quantitatively synthesize the diagnostic performance of ML–based models for the preoperative prediction of PA consistency. The study followed the “Preferred Reporting Items for Systematic Reviews and Meta-Analyses” (PRISMA) guidelines [29]. The study was not registered in any registries.

### Search strategy

A comprehensive search was conducted in PubMed, Embase, Scopus, and Web of Science from inception to September 11, 2025. Search terms combined controlled vocabulary and free-text words for “pituitary adenoma,” “machine learning,” and “tumor consistency” using Boolean operators. The full search strategies for each database are provided in Supplementary Table S1. No language or publication restrictions were applied.

### Eligibility criteria

The PICO framework of the current study is summarized in Supplementary Table S2. Studies were included if they developed or validated ML–based models using MRI for the preoperative prediction of PA consistency. Eligible studies enrolled patients with histologically or intraoperatively confirmed PAs and reported at least one diagnostic performance metric, area under the curve (AUC), accuracy (ACC), sensitivity (SEN), and specificity (SPE), for model evaluation. Both retrospective and prospective observational studies were included, provided that the ML model underwent internal or external validation.

Studies were excluded if they were reviews, meta-analyses, conference abstracts, editorials, letters, case reports, animal experiments, or phantom imaging studies. Investigations that did not apply ML, deep learning (DL), or neural network (NN) algorithms, such as those using only conventional logistic regression (LR) or classical statistical analyses without algorithmic training or feature selection, were excluded. Studies relying solely on qualitative MRI features or signal intensity without ML integration, lacking intraoperative or pathological ground truth for tumor consistency, or failing to report at least one quantitative diagnostic performance metric were also excluded. When overlapping datasets were identified, the most comprehensive or most recent publication was retained.

### Study selection process, data extraction, and risk of bias assessment

Following the literature search, all identified records were imported into Covidence for reference management and screening. Duplicate records were automatically removed. Two independent reviewers performed the title and abstract screening, and any disagreements were resolved by a third reviewer. The same procedure was applied during the full-text review to determine final eligibility according to predefined inclusion and exclusion criteria. Studies meeting the eligibility criteria were included for data extraction. The complete list of extracted variables is presented in Supplementary Table S3, encompassing study characteristics, MRI acquisition parameters, radiomics workflow, ML model features, validation methods, and diagnostic performance outcomes. The definitions of the evaluated outcomes, including AUC, ACC, SEN, and SPE, are summarized in Supplementary Table S4. Data extraction was conducted independently by two reviewers and cross-checked to ensure accuracy and consistency. The Quality Assessment of Diagnostic Accuracy Studies 2 (QUADAS-2) tool was used to assess the risk of bias (RoB) and applicability concerns across four domains: patient selection, index test, reference standard, and flow and timing [41]. Assessments were performed independently by two reviewers, and discrepancies were resolved through discussion and consensus.

### Statistical analysis

The meta, metafor, and mada packages were used in R software (version 4.4.2) for statistical analysis. For each model, we selected the highest reported AUC for PA consistency prediction, prioritizing values from external or independent test sets when available. If not reported, the highest value from internal validation or training sets was used. Pooled estimates of AUC and ACC were computed using inverse-variance random-effects meta-analysis, and 95% confidence intervals (CIs) for ACC and AUC were obtained using the Wilson score interval and the Hanley and McNeil method when CIs were not provided. For binary diagnostic accuracy measures (SEN and SPE), event counts (true positives, false negatives, true negatives, false positives) were reconstructed from reported SEN, SPE, prevalence, and sample size. Proportions were pooled using logit transformation (PLOGIT) with the Clopper–Pearson method for CIs under a random-effects model. Diagnostic odds ratios (DORs) were calculated with the metabin function using random-effects modeling. Summary receiver operating characteristic (SROC) curves were fitted with the Reitsma bivariate model implemented in the mada package. Heterogeneity was assessed using the I^2^ statistic according to Cochrane thresholds. Sensitivity analyses were performed using a leave-one-out approach for all primary outcomes to evaluate the influence of individual studies on pooled estimates. Publication bias was examined using Egger’s regression test, and funnel plots were visually inspected when more than ten studies were available. When asymmetry was observed, the trim-and-fill method was applied to estimate the number of potentially missing studies and to adjust pooled effect sizes. Significant moderators were identified using *p* < 0.05.

## Results

### Study selection process

**Fig. 1:**
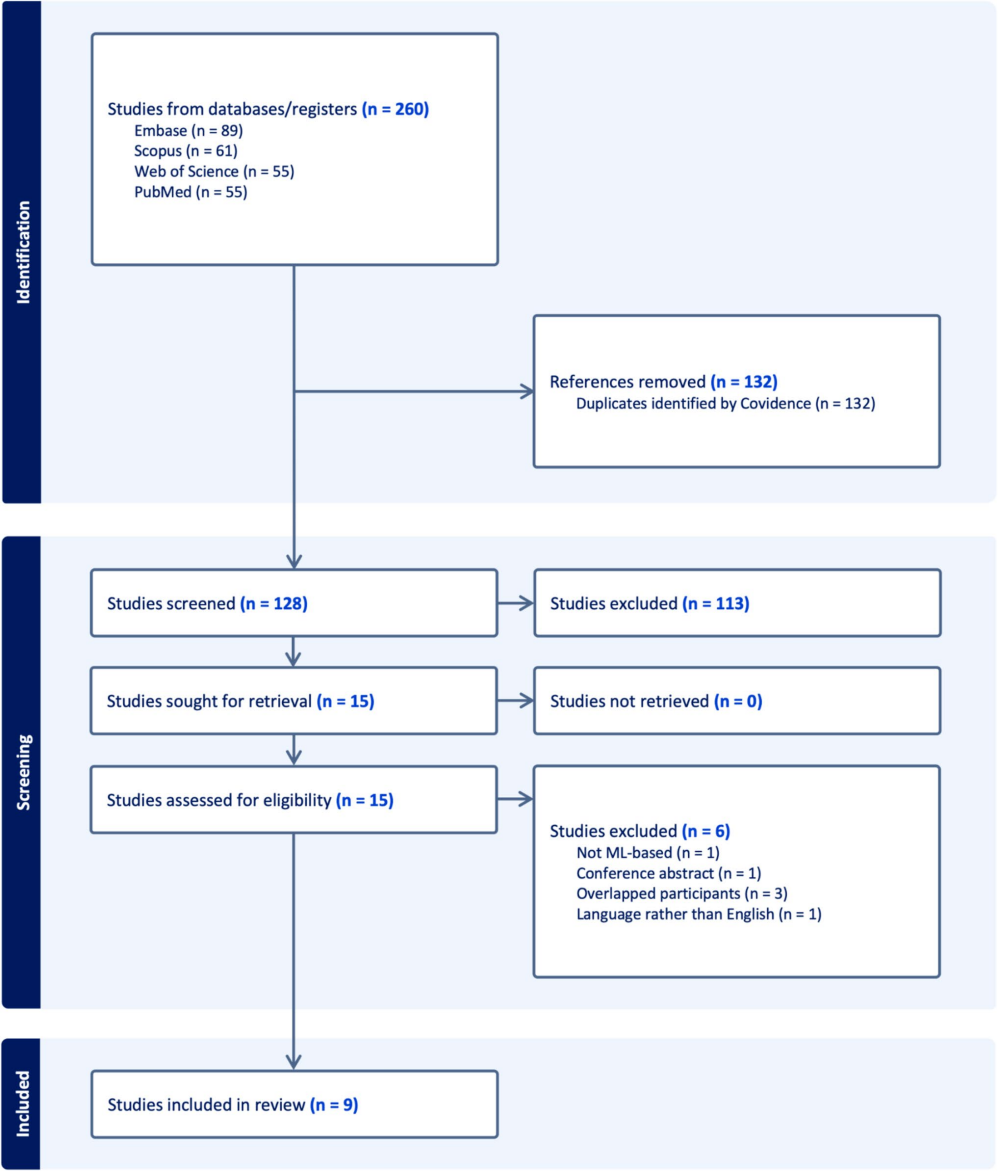
PRISMA flowchart of the included studies

The initial database search across PubMed, Embase, Scopus, and Web of Science yielded 260 unique records (PubMed = 55, Embase = 89, Scopus = 61, Web of Science = 55) (Fig. 1). After removing 132 duplicates automatically identified by Covidence, 128 studies remained for title and abstract screening. Of these, 113 were excluded for irrelevance to ML–based prediction of PA consistency. The remaining 15 articles underwent full-text review, during which six were excluded for the following reasons: not ML-based (*n* = 1), conference abstract without full data (*n* = 1), overlapping study populations (*n* = 3), and non-English language (*n* = 1) [7, 14, 15, 42]. Ultimately, nine studies met all eligibility criteria and were included in the qualitative synthesis and quantitative meta-analysis [6, 8, 11, 24, 27, 32, 39, 40, 45]. No studies were excluded due to retrieval failure or inaccessible full text.

### Risk of bias assessment

The overall quality assessment revealed a moderate to high risk of bias across most included studies (Supplementary Table S5). While several analyses demonstrated low concern about the reference standard, many showed unclear or high risks in the patient selection and index test domains, reflecting variability in recruitment design, feature extraction methods, and model validation strategies. The predominance of retrospective single-center designs and insufficient reporting of blinding procedures further limited the assessment of internal validity. Collectively, these findings indicate that although the included studies provide valuable preliminary evidence, methodological heterogeneity and incomplete reporting reduce the overall reliability and generalizability of their results.

### Baseline characteristics and model outcomes

Across the nine included studies, a total of 1,621 patients with PA were analyzed, with individual sample sizes ranging from 52 to 542 cases (Table 1). The studies were published from 2019 to 2025. The average age ranged from 38.7 to 53.5 years, and 51.3% (761/1,484) were female. The cohort included 56.8% (707/1,244) with non-functional PA and 43.2% (537/1,244) with functional PA.

**Table 1:** Baseline and demographic characteristics

Study	Recruitment Period	No. of Patients	Mean Age (y)	Gender (M/F)	Adenoma Size (micro/macro)	Functional Status (FPA/NFPA)	Prior Surgery	Prior Radiation	Surgical Approach
Cao et al. 2025 [6]	2018–2023	137	50.4	NA/NA	NA/NA	NA/NA	NA	NA	NA
Cuocolo et al. 2020 [11]	2013–2017	89	52.2	51/38	0/89	25/64	0	0	Endoscopic endonasal transsphenoidal
Liang et al. 2025 [24]	2017–2022	350	51.9	174/176	0/350	219/131	0	0	Endoscopic transsphenoidal surgery
Mendi et al. 2023 [27]	NA	52	49.5	30/22	NA/NA	40/12	0	0	Transnasal transsphenoidal (51/52); 1 transcranial
Pereira et al. 2025 [32]	2015–2021	70	51.1	37/33	0/70	NA/NA	0	0	Endoscopic endonasal transsphenoidal
Wan et al. 2022 [40]	2012–2015	156	NA	71/85	0/156	32/124	0	0	Transsphenoidal: 138; Craniotomy: 18
Wang et al. 2021 [39]	NA	170	38.7	63/107	NA/NA	NA/NA	NA	NA	Transsphenoidal
Zeynalova et al. 2019 [45]	2009–2017	55	50.1	31/24	0/55	15/40	0	0	Transsphenoidal
Černý et al. 2025 [8]	2008–2018	542	53.5	266/276	0/542	206/336	0	0	Endoscopic endonasal approach

*FPA* Functioning Pituitary Adenoma, *NFPA* Non-Functioning Pituitary Adenoma, *NA* Not Available

Regarding imaging features, the MRI field strength was 1.5 T or 3.0 T in all studies, and the slice thickness ranged from 2.5 to 5 mm (Table 2). The tumor diameter was reported between 22 and 29 mm, with available tumor volume data around 7–8 cm^3^ in selected studies. Tumor consistency was primarily determined by intraoperative surgeon grading. All studies used binary classification schemes (“soft” versus “firm/hard”), with the firm category generally defined as requiring sharp dissection or non-suctionable texture. Segmentation was manual or semi-automated in 78% of studies, with preprocessing steps—such as bias-field correction, z-score normalization, and isotropic resampling, being consistently applied.

**Table 2:** Imaging and consistency parameters

Study	Mean Max Diameter (mm)	Mean Tumor Volume (cc)	MRI Field Strength	Slice Thickness (mm)	Ground Truth Source	Consistency Scale Name	Consistency Binarization Rule	Class Marked Positive	Positive N	Negative N	Preprocessing Steps	Registration	ROI Definition	Segmentation Method	Segmentation Tool
Cao et al. 2025 [6]	NA	NA	3.0 T	NA	Biopsy + clinician labeling	Binary: soft vs. firm	Soft vs. Firm	Hard texture	47	90	Signal intensity normalization, registration to 1-mm isovoxel, resampling	Yes	Whole tumor	NA	NA
Cuocolo et al. 2020 [11]	25	NA	1.5 T or 3.0 T	NA	Intraoperative surgeon scale	Binary (soft vs fibrous)	Soft vs. Fibrous	Fibrous	21	68	Resample (2 × 2 × 2 mm), intensity normalization (z-score), fixed bin width (= 3), Laplacian of Gaussian filters (σ = 2.0–3.5), wavelet decompositions	Yes	2D ROI on coronal slice of maximum tumor extension	Manual	ITK-SNAP v3.8.0
Liang et al. 2025 [24]	NA	NA	1.5 T or 3.0 T	NA	Intraoperative surgeon scale	Binary: soft vs firm	Soft vs. Firm	Firm tumor	90	260	N4 bias-field correction, Z-score normalization, resample to 1 × 1 × 1 mm^3^	Yes	Whole tumor	Semi-Segmentation	3D Slicer
Mendi et al. 2023 [27]	NA	NA	3.0 T	2.50	Intraoperative surgeon scale	Binary: soft vs. firm	Soft vs. Firm	Firm tumor	20	32	Standardization; resampling (0.7 × 0.7 × 5 mm^3^); gray-level discretization 64 bins	No	Whole tumor	Manual	IBEX Software
Pereira et al. 2025 [32]	29	NA	NA	NA	Intraoperative surgeon assessment + pathology confirmation	Binary: soft vs. firm	Soft vs. Non-soft	Non-soft consistency	11	59	One-hot encoding; PCA for dimension reduction; missing data handled by chained equations and KNN imputation	No	Whole tumor	Manual	Standard MRI
Wan et al. 2022 [40]	NA	NA	3.0 T	3	Intraoperative surgeon scale	Binary: soft vs. firm	Soft vs Hard	Hard consistency	52	104	Bias-field correction (N4), intensity normalization, resampling	Yes	Whole tumor	Automated	MATLAB
Wang et al. 2021 [39]	NA	7.8	3.0 T	3	Intraoperative surgeon scale	Binary: soft vs. firm	Soft vs. Firm	Firm tumor	59	111	Normalization, feature selection (PyRadiomics + ANOVA F-test); segmentation preprocessing (contrast-enhanced T1 only)	No	Whole tumor	Manual	ITK-SNAP
Zeynalova et al. 2019 [45]	24.1	NA	1.5 T	2.50	Surgical notes + intraoperative impression, histopathology	Binary: soft vs. firm	Soft vs hard	Hard tumor	13	42	N4 bias field correction, ± 3 sigma normalization, resampling to 1 × 1 mm^2^, discretization bin-width 0.06	No	Solid tumor, excluding peripheral tissue	Manual	PyRadiomics
Černý et al. 2025 [8]	22.1	NA	1.5 T or 3.0 T	4.89	Intraoperative surgeon assessment	Binary: soft vs. firm	Firm vs. Soft	Firm tumor	122	420	Bias-field correction, variance & correlation filtering of radiomic features	Yes	Whole tumor	Automated	AI-based model, not manual ITK-SNAP

*ROI* Region of Interest, *MRE* Magnetic Resonance Elastography, *NA* Not Available

Among the included studies, 77.8% (6/9) were ML-based, 11.1% (1/9) were DL-based, and 11.1% (1/9) were NN-based (Table 3). Validation techniques included tenfold cross-validation in 55.6% (5/9), train-test split in 33.3% (3/9), and fivefold cross-validation in 11.1% (1/9). The algorithms used across the studies included Extra Trees Classifier (ET), Random Forest (RF), Support Vector Machine (SVM), logistic regression (LR), k-Nearest Neighbors (kNN), Artificial Neural Network (ANN), and DL architectures combining U-Net feature extractors with Convolutional Recurrent Neural Networks (CRNN). The reported diagnostic performance was consistently high, with AUC ranging from 0.71 to 0.99, accuracy from 0.73 to 0.95, SEN from 0.66 to 1.00, and SPE from 0.79 to 0.90.

**Table 3:** Machine learning model characteristics and performance

Study	AI Model (ML/DL/NN)	Validation Method (Train–Test/K-fold/External)	Data Type (Radiomics/Genomic/Clinical)	Image Sequence Used	Best Predictor — Algorithm	AUC (test/validation)	ACC (test/validation)	SEN/Recall (test/validation)	SPE (test/validation)
Cao et al. 2025 [6]	DL	Train–Test split (80:20)	Imaging	T1WI, T2WI, and multi-sequence	Improved U-Net feature extractor + CRNN classifier	NA	0.9464	NA	NA
Cuocolo et al. 2020 [11]	ML	fivefold CV	Radiomics	T2WI	ET	0.990	0.9300	1	0.8700
Liang et al. 2025 [24]	ML	Train–Test split (70:30)	Radiomics + Clinical	T1WI, T2WI, CE-T1WI	LR	0.913	0.8400	0.8850	0.8250
Mendi et al. 2023 [27]	ML	tenfold CV	Radiomics	T1W and T2W	SVM	0.956	NA	0.6558	0.8367
Pereira et al. 2025 [33]	ML	tenfold CV	Imaging + Clinical	T1/T2, diffusion-weighted MRI (ADC)	SVM	0.833	NA	0.7310	0.8980
Wan et al. 2022 [40]	ML	tenfold CV	Radiomics	T1WI, T1CE, T2WI	RF	0.900	0.8700	0.8300	0.8700
Wang et al. 2021 [39]	ML	tenfold CV	Radiomics + Clinical	T1CE	KNN	0.920	0.8180	NA	NA
Zeynalova et al. 2019 [45]	ANN	tenfold CV	Radiomics	T2WI	ANN	0.710	0.7250	0.6580	0.7860
Černý et al. 2025 [8]	ML	Train–Test split (70:30)	Radiomics	T2WI	RF	NA	0.8160	NA	NA

*ML* Machine Learning, *DL* Deep Learning, *NN* Neural Network, *AUC* Area Under the Curve, *ACC* Accuracy, *SEN* Sensitivity, *SPE* Specificity, *NA* Not Available

### Meta-analysis of outcomes

**Fig. 2:**
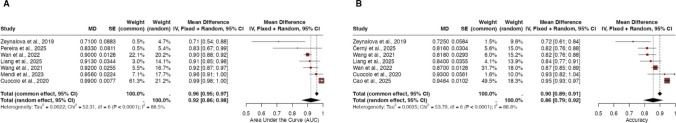
Meta-analyses of pooled model performance showing (**A**) area under the curve and (**B**) accuracy across studies

**Fig. 3:**
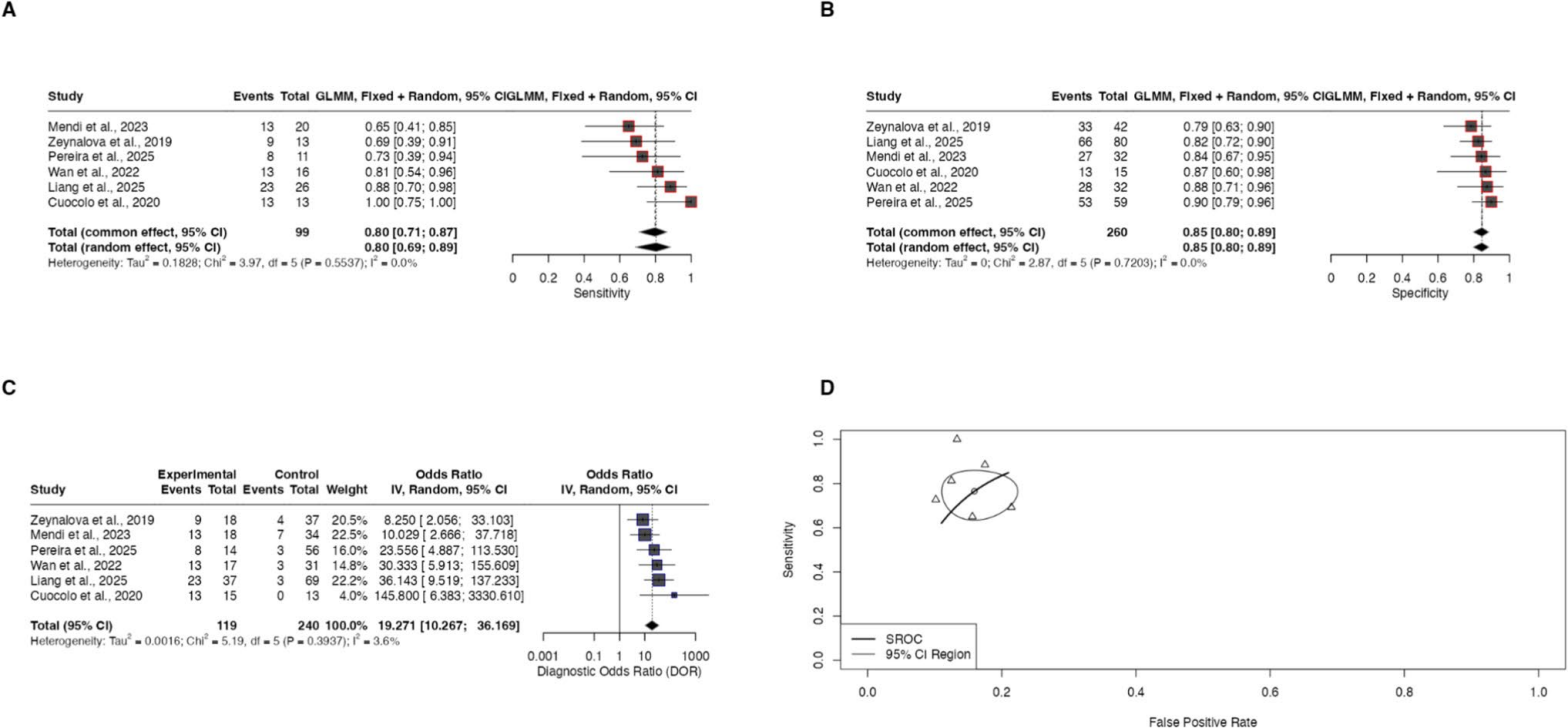
Diagnostic accuracy synthesis showing pooled (**A**) sensitivity, **B** specificity, **C** diagnostic odds ratio, and (**D**) SROC curve from the bivariate Reitsma model

Based on seven studies included in the meta-analysis, the pooled AUC was 0.92 (95% CI: 0.86–0.98) under a random-effects model, confirming excellent overall discriminative ability (Fig. 2A). The pooled ACC across studies was 0.86 (95% CI: 0.79–0.92) (Fig. 2B). In terms of diagnostic proportions, the pooled SEN reached 0.80 (95% CI: 0.71–0.87) and the pooled SPE was 0.85 (95% CI: 0.80–0.89) (Fig. 3A-B). The pooled DOR was 19.27 (95% CI: 10.27–36.17), corresponding to approximately 19 times higher odds of correct classification compared with incorrect predictions (Fig. 3C). The SROC analysis using a bivariate Reitsma model yielded an overall SROC AUC of 0.878 and a partial AUC of 0.741, confirming strong discriminative capacity across sensitivity–specificity trade-offs (Fig. 3D). The summary false positive rate (FPR) was 0.16 (95% CI: 0.12–0.21).

### Sensitivity analysis

The sensitivity analysis demonstrated that the pooled results were highly stable and internally consistent across all performance measures. Excluding any single study did not meaningfully affect the summary estimates of AUC, ACC, SEN, SPE, or DOR. While minor variations were observed, the overall diagnostic performance remained uniform, indicating that no study exerted disproportionate influence on the meta-analytic findings. These results confirm the robustness and reproducibility of the pooled outcomes and reinforce the reliability of the overall conclusions.

### Publication bias

Egger’s test revealed no significant evidence of publication bias across all pooled outcomes. The p-values were 0.0941 for AUC, 0.0950 for ACC, 0.0920 for SEN, 0.3305 for SPE, and 0.1701 for DOR, all exceeding the 0.05 significance threshold. While AUC, ACC, and SEN displayed marginal p-values near 0.09, these do not indicate significant small-study effects and are more likely attributable to between-study variability rather than selective reporting. Collectively, these findings confirm that the meta-analytic results are stable and not meaningfully affected by publication bias.

### GRADE assessment

Using the GRADE framework, the certainty of evidence for each pooled outcome was as follows. For AUC, the evidence was rated as moderate due to consistent direction of effect but downgraded for heterogeneity among algorithms. For ACC, the certainty was moderate, supported by stable pooled estimates but limited by retrospective study designs. For SEN, the evidence was moderate to high because of consistent findings and negligible heterogeneity. For SPE, the certainty was high, given its uniform estimates and low between-study variability. For DOR, the evidence was moderate, reflecting strong diagnostic strength but limited generalizability due to small sample sizes. Overall, the GRADE assessment indicates that ML-based models show moderate to high confidence in predicting PA consistency, though prospective multicenter validation is needed to reach high-certainty evidence.

## Discussion

This meta-analysis combined all available ML–based studies on preoperative prediction of PA consistency and demonstrated strong pooled diagnostic performance across multiple metrics. The combined estimates showed an AUC of 0.92 (95% CI 0.86–0.98), accuracy of 0.86 (95% CI 0.79–0.92), sensitivity of 0.80 (95% CI 0.71–0.87), specificity of 0.85 (95% CI 0.80–0.89), and a DOR of 19.27 (95% CI 10.27–36.17). These findings indicate that ML-based models correctly differentiate firm from soft adenomas in nearly nine of ten cases, with approximately 19-fold greater odds of correct classification compared with chance prediction.

Preoperative identification of PA consistency is clinically important because firm or fibrous tumors substantially increase surgical difficulty, often requiring extracapsular dissection, extended endonasal approaches, ultrasonic aspiration, or even conversion to a transcranial route when safe progression is not possible [1–5]. Firm tumors are also independently associated with markedly lower gross total resection rates and a higher likelihood of reoperation compared with soft tumors, reflecting reduced internal decompression capacity and stronger collagenous adhesion to adjacent neurovascular structures [6–9]. Beyond the extent of resection, firm texture confers increased postoperative risks, including cerebrospinal fluid leak, transient diabetes insipidus, cranial nerve deficits, and delayed hyponatremia, making preoperative prediction essential for perioperative risk counselling and patient expectation management [1, 10]. Knowledge of consistency also guides operative duration planning and resource allocation, ensuring the availability of advanced instruments and anticipating scenarios where subtotal resection followed by adjuvant therapy may be preferable [2, 11]. Therefore, ML-based models for consistency prediction offer meaningful value by informing surgical strategy, reducing avoidable morbidity, and optimizing postoperative follow-up pathways [3, 7].

From a clinical perspective, the excellent pooled AUC and DOR emphasize the promise of ML algorithms as noninvasive surrogates for intraoperative texture grading, potentially improving surgical preparedness and risk assessment. Accurate preoperative identification of firm or fibrous adenomas could facilitate operative planning, selection of surgical tools, and patient counseling. Together, the pooled estimates reinforce that ML-based modeling can provide objective, reproducible, and clinically actionable information in the preoperative evaluation of pituitary tumors.

Recent studies have provided additional quantitative evidence that reinforces and extends our pooled findings. Zeynalova et al. analyzed 55 patients (13 firm vs 42 soft adenomas) using a simple first-order histogram-based ANN and achieved an AUC of 0.71 with an ACC of 72.5%, notably outperforming the conventional T2 signal-intensity ratio method (AUC = 0.55). Their results were the first to demonstrate that voxel-wise intensity distribution, rather than mean T2 signal, carries predictive information related to intratumoral collagen density [45]. Cuocolo et al. expanded this concept in 89 patients (68 soft, 21 fibrous), extracting more than 1,100 texture features from T2-weighted MRI and identifying 14 stable variables through recursive feature elimination. Using an ET classifier, they reported an AUC of 0.99, an accuracy of 93%, an SEN of 100%, and an SPE of 87%, highlighting how rigorous feature-stability filtering can yield near-perfect internal discrimination, albeit with potential overfitting given the single-center dataset [11].

Multiparametric and multi-sequence radiomics have also shown superior generalizability. Wan et al. retrospectively evaluated 156 macroadenomas (104 soft, 52 hard) using combined T1WI/T1CE/T2WI sequences and RF and SVM classifiers. The combined model achieved an AUC of 0.90, an ACC of 0.87, an SEN of 0.83, and an SPE of 0.87, surpassing all single-sequence models by approximately 0.08 in AUC [40]. Similarly, the dual-center study by Liang et al. included 350 patients and compared LR and RF combinations of clinicoradiologic and radiomic features. Their best model achieved an AUC of 0.913 and an accuracy of 0.84 in the external test cohort, with SHAP analysis showing that wavelet- and Laplacian-filtered T2WI features contributed more than 60% of the model's importance. These findings collectively demonstrate that combining multiple MRI contrasts with interpretable machine-learning frameworks improves both robustness and clinical transparency [24].

Other studies incorporated non-imaging variables and clinical outcomes, underscoring the biological and prognostic implications of radiomic firmness. Pereira et al. utilized demographic parameters and MRI metrics in 70 patients. They found that an SVM classifier achieved an AUC of 0.83 and an F1 score of 0.63, with model explainability indicating that male sex and age ≤ 42 years were the dominant predictors of non-soft consistency [32]. In a large-scale study involving 542 patients, Černý et al. demonstrated that firm tumors were linked to significantly lower gross total resection rates (35.2% vs. 67.1%; *p* < 0.001), and a radiomics-based RF model predicted tumor firmness with 81.6% ACC [8]. Finally, Cao et al. introduced a fully automated 3D DL architecture combining U-Net and convolutional recurrent layers across glioma and pituitary datasets, achieving 94.6% ACC for pituitary tumor texture classification without handcrafted feature extraction [6]. Taken together, these studies show a steady methodological evolution, from early histogram descriptors with modest accuracy to recent interpretable, multiparametric, and DL frameworks consistently reaching AUC values more than 0.90, reflecting substantial progress toward clinically deployable, quantitative tools for preoperative assessment of PA consistency.

ML, DL, and NN–based models, despite their impressive predictive abilities, face several core limitations. They are naturally dependent on data and require large, high-quality, and well-annotated datasets, something often difficult to obtain in medical imaging [3, 12, 13]. Their performance can decline significantly when applied to data from different scanners, institutions, or patient populations due to the “domain shift” problem, which limits their generalizability [4, 5]. Many models function as “black boxes,” offering limited interpretability and explainability, which reduces clinical trust and impedes regulatory approval [21, 25, 26]. Overfitting is common, especially when models are trained on small or homogeneous samples, leading to inflated internal performance that fails under real-world testing [2]. Furthermore, algorithmic bias can arise from unbalanced data, potentially reinforcing disparities in patient care [3, 12, 13, 31, 43]. DL and NN architectures are also computationally intensive, requiring substantial hardware, energy, and expertise to develop and deploy [10, 36]. Finally, the lack of standardization in model reporting, feature extraction, and validation frameworks complicates the reproducibility and clinical translation of results. These limitations collectively emphasize that ML, DL, and NN models should complement, rather than replace, expert clinical judgment until robust, transparent, and externally validated systems become widely available.

ML–based models demonstrate strong potential for clinical integration in the preoperative assessment of PAs. Their high pooled diagnostic performance (AUC = 0.92, ACC = 0.86, SEN = 0.80, SPE = 0.85) suggests that these algorithms can serve as noninvasive tools to estimate tumor firmness and guide surgical planning. By identifying firm or fibrous adenomas preoperatively, surgeons can better anticipate intraoperative challenges, select appropriate dissection techniques, and counsel patients regarding operative risks and expected outcomes. Incorporating validated ML models into radiology workflows, either as adjunct decision-support systems or embedded modules in PACS, could improve surgical preparedness and facilitate personalized operative strategies.

This meta-analysis is limited by the small number of available studies and the predominance of retrospective, single-center designs. The included models exhibited methodological heterogeneity in MRI protocols, segmentation techniques, and ground-truth definitions of tumor consistency, which may have contributed to between-study variability. All included radiomics studies used manual segmentation of the PA on preoperative MRI, most often on T2-WI, followed by standard radiomics workflows for dimensionality reduction. Notably, none of the studies performed voxel-resampling, and all high-performing models were developed using native voxel dimensions, so no clear trend toward improved performance with resampling could be evaluated. Across studies, manual segmentation consistently produced reproducible features and achieved higher predictive performance than earlier semi-quantitative T2-signal methods. Many algorithms lacked external validation, reducing generalizability across institutions and imaging platforms. Furthermore, incomplete reporting of feature-selection pipelines and cross-validation procedures restricted reproducibility. The potential for overfitting, publication bias toward positive results, and limited interpretability of complex models such as deep neural networks also remain significant constraints that warrant cautious clinical translation. Because all eligible studies evaluated only macroadenomas, our pooled estimates do not include microadenomas or giant adenomas. This size-restricted evidence base limits the generalizability of radiomics performance across the full spectrum of PA sizes. The available studies showed considerable methodological heterogeneity, with variation in radiomics pipelines, ML models, and overall risk of bias. Although we used a random-effects model, these differences cannot be fully controlled for, and the pooled AUC should be interpreted cautiously as an overall signal rather than a precise performance estimate.

Future research should focus on large, prospective, multicenter studies employing standardized MRI acquisition, radiomic feature extraction, and consistency grading to enhance reproducibility and external validity. Development of transparent and interpretable ML frameworks with open-source datasets and harmonized reporting standards will be essential for regulatory approval and clinical adoption. Integration of radiomic, clinical, and biochemical features into multimodal predictive models may further improve accuracy and biological insight. Ultimately, integrating ML tools into real-time surgical navigation or preoperative planning platforms could facilitate dynamic, patient-specific guidance for pituitary surgery, thereby bridging the gap between algorithmic performance and clinical utility.

## Conclusion

ML–based models demonstrate excellent pooled diagnostic accuracy for preoperative prediction of pituitary adenoma consistency, with a combined AUC of 0.92, ACC of 0.86, SEN of 0.80, and SPE of 0.85. These findings underscore the capacity of ML algorithms to noninvasively characterize tumor firmness, offering valuable information for surgical preparation and risk stratification. Despite current limitations related to dataset heterogeneity, limited external validation, and lack of standardization, the consistent diagnostic strength across studies highlights the readiness of these models for translational development. Continued multicenter, prospective validation with harmonized radiomic pipelines and interpretable algorithmic frameworks will be pivotal to enable clinical deployment and integration into neurosurgical decision support systems.

## Supplementary Information

Below is the link to the electronic supplementary material.

ESM 1 Supplementary Material 1 (DOCX 22.9 KB)

ESM 2 Supplementary Material 2 (DOCX 417 KB)

## Data Availability

“The data supporting this study's findings are available from the corresponding author upon reasonable request.”
