# Supplementary material for: Machine learning-based models for preoperative prediction of pituitary adenoma consistency: a systematic review and meta-analysis
Source: Acta Neurochir (Wien). 2026 Jan 24;168(1):23. doi: 10.1007/s00701-026-06775-w (PMC12835045; doi:10.1007/s00701-026-06775-w)
Supplement: Supplementary file 2 — Supplementary Material 2 (DOCX 417 KB) [file 701_2026_6775_MOESM2_ESM.docx]

Supplementary File

Machine Learning-Based Models for Preoperative Prediction of Pituitary Adenoma Consistency: A Systematic Review and Meta-Analysis

| Supplementary Table S1: Search strategies for various databases. | |
| --- | --- |
| Database | Search strategy |
| PubMed | (("Pituitary Neoplasms"[Mesh] OR "ACTH-Secreting Pituitary Adenoma"[Mesh] OR "Growth Hormone-Secreting Pituitary Adenoma"[Mesh] OR "Prolactinoma"[Mesh] OR (Pituitary[tiab] AND (Neoplasm*[tiab] OR Adenoma*[tiab])) OR (Pituitary[tiab] AND (macroadenoma*[tiab] OR microadenoma*[tiab])) OR "cushing disease"[tiab] OR "cushing"[tiab] OR "pituitary acth hypersecretion"[tiab] OR "acth hypersecretion"[tiab] OR "hyperpituitarism"[tiab] OR "prolactinoma"[tiab] OR "gonadotroph adenomas"[tiab] OR "gonadotroph tumor"[tiab] OR "gonadotroph tumours"[tiab] OR "gonadotropic adenoma"[tiab] OR "gonadotropinoma"[tiab] OR "thyrotroph adenoma"[tiab] OR "TSHoma"[tiab] OR "GH secreting adenoma"[tiab] OR "acromegaly"[tiab] OR "Acromegaly"[Mesh]) AND ("Artificial Intelligence"[Mesh] OR "Machine Learning"[Mesh] OR "Deep Learning"[Mesh] OR radiomic*[tiab] OR "machine learning"[tiab] OR "deep learning"[tiab] OR "artificial intelligence"[tiab] OR "transfer learning"[tiab] OR "ensemble learning"[tiab] OR "computer vision"[tiab] OR "artificial neural networks"[tiab] OR "convolutional neural network"[tiab]) AND ("consistency"[tiab] OR "tumor consistency"[tiab] OR "tumour consistency"[tiab] OR "lesion consistency"[tiab] OR "intraoperative consistency"[tiab] OR fibrous[tiab] OR firm[tiab] OR hard[tiab] OR soft[tiab] OR gelatinous[tiab] OR cystic[tiab] OR solid[tiab] OR texture[tiab])) |
| Embase | (('pituitary tumor'/exp OR 'pituitary adenoma'/exp OR 'pituitary neoplasm'/exp OR (pituitary:ti,ab AND (adenoma*:ti,ab OR neoplasm*:ti,ab OR macroadenoma*:ti,ab OR microadenoma*:ti,ab)) OR 'cushing disease':ti,ab OR 'acromegaly'/exp OR acromegaly:ti,ab OR 'gh secreting adenoma':ti,ab OR prolactinoma:ti,ab OR 'gonadotroph adenoma':ti,ab) AND ('artificial intelligence'/exp OR 'machine learning'/exp OR 'deep learning'/exp OR radiomic*:ti,ab OR 'machine learning':ti,ab OR 'deep learning':ti,ab OR 'artificial intelligence':ti,ab OR 'transfer learning':ti,ab OR 'ensemble learning':ti,ab OR 'computer vision':ti,ab OR 'artificial neural network':ti,ab OR 'convolutional neural network':ti,ab) AND ('consistency':ti,ab OR 'tumor consistency':ti,ab OR 'tumour consistency':ti,ab OR 'lesion consistency':ti,ab OR 'intraoperative consistency':ti,ab OR fibrous:ti,ab OR firm:ti,ab OR hard:ti,ab OR soft:ti,ab OR gelatinous:ti,ab OR cystic:ti,ab OR solid:ti,ab OR texture:ti,ab)) |
| Scopus | (TITLE-ABS-KEY(pituitary AND (adenoma* OR neoplasm* OR macroadenoma* OR microadenoma* OR "cushing disease" OR acromegaly OR prolactinoma OR "gh secreting adenoma" OR "gonadotroph adenoma")) AND TITLE-ABS-KEY("machine learning" OR "deep learning" OR "artificial intelligence" OR radiomic* OR "transfer learning" OR "ensemble learning" OR "computer vision" OR "artificial neural network" OR "convolutional neural network") AND TITLE-ABS-KEY(consistency OR "tumor consistency" OR "tumour consistency" OR "lesion consistency" OR "intraoperative consistency" OR fibrous OR firm OR hard OR soft OR gelatinous OR cystic OR solid OR texture)) |
| WOS | (TS=(pituitary AND (adenoma* OR neoplasm* OR macroadenoma* OR microadenoma* OR "cushing disease" OR acromegaly OR prolactinoma OR "gh secreting adenoma" OR "gonadotroph adenoma")) AND TS=("machine learning" OR "deep learning" OR "artificial intelligence" OR radiomic* OR "transfer learning" OR "ensemble learning" OR "computer vision" OR "artificial neural network" OR "convolutional neural network") AND TS=(consistency OR "tumor consistency" OR "tumour consistency" OR "lesion consistency" OR "intraoperative consistency" OR fibrous OR firm OR hard OR soft OR gelatinous OR cystic OR solid OR texture)) |
| September 11, 2025 | |

| Supplementary Table S2. PICO framework | |
| --- | --- |
| Population (P) | Patients with pituitary adenomas |
| Intervention (I) | Development and application of machine learning–based models (e.g., radiomics, genomics, transcriptomics, proteomics, multimodal data integration) for biomarker prediction |
| Comparison (C) | Not applicable (no direct intervention comparator); conventional statistical methods or clinical/imaging assessment may be reported in some studies |
| Outcome (O) | Diagnostic/predictive performance of ML models for biomarkers (e.g., Ki-67 index, PIT1, SF1, TPIT, p53, VEGF, EGFR, MGMT, microRNAs, DNA methylation, hormonal markers); metrics include AUC, accuracy, sensitivity, specificity |

| Supplementary Table S3. Extracted variables | |
| --- | --- |
| Baseline | Outcome |
| Digital Object Identifier (DOI)  Study (First author and publication year)  Country of study population  Collaborating institutions  Recruitment period  Total number of patients  Mean age (years)  Number of male patients  Number of female patients  Number of microadenomas (<10 mm)  Number of macroadenomas (≥10 mm)  Number of functioning pituitary adenomas  Number of non-functioning pituitary adenomas  Number of patients with prior surgery  Number of patients with prior radiotherapy  Surgical approach (endoscopic, transcranial, or combined)  Mean maximal tumor diameter (mm)  Mean tumor volume (cm³)  MRI field strength (1.5 T, 3 T, or other)  MRI slice thickness (mm)  Source of ground truth (intraoperative observation, surgeon scale, pathology, or MRE)  Consistency grading scale (e.g., soft/intermediate/firm; three-grade; binary)  Binarization rule for consistency (e.g., soft vs firm; soft + intermediate vs firm)  Definition of positive class (e.g., firm consistency)  Number of positive cases  Number of negative cases  Image preprocessing steps (bias-field correction, skull stripping, N4, z-normalization, resampling)  Image registration (yes/no; target space if applicable)  Region of interest definition (whole tumor, solid component only, exclusion of cystic/hemorrhagic areas)  Segmentation method (manual, semi-automated, automated)  Segmentation software (e.g., ITK-SNAP, 3D Slicer, MATLAB, in-house tool) | Purpose of the model (e.g., consistency prediction, resection extent, radiomic interpretation) Artificial intelligence model type (machine learning, deep learning, neural network, hybrid)  Validation strategy (train–test split, k-fold cross-validation, external validation)  Type of input data (radiomic, clinical, genomic, multimodal)  MRI sequences used (e.g., T1-weighted, contrast-enhanced T1-weighted, T2-weighted, diffusion-weighted, multiparametric MRI)  Algorithm of best-performing predictive model  Area under the receiver operating characteristic curve (AUC) for test or validation set  95% confidence interval (lower and upper bounds) for AUC  Accuracy of the best predictive model (test or validation set)  95% confidence interval (lower and upper bounds) for accuracy  Sensitivity (recall) of the best predictive model  Specificity of the best predictive model  Prevalence of the positive class in the dataset  Total number of positive and negative cases |
|  |  |
|  |  |

| Supplementary Table S4. Definition of outcomes | |
| --- | --- |
| Area Under the Receiver Operating Characteristic Curve | A global measure of a model’s discriminative ability to correctly distinguish between positive (firm) and negative (soft) classes across all possible thresholds. |
| Accuracy | The proportion of all correctly classified observations (both positive and negative) among all evaluated samples. |
| Sensitivity | The ability of the model to correctly identify positive cases (e.g., firm adenomas correctly predicted as firm). |
| Specificity | The ability of the model to correctly identify negative cases (e.g., soft adenomas correctly predicted as soft). |
| Diagnostic Odds Ratio | A single indicator combining sensitivity and specificity that represents the odds of a correct classification relative to an incorrect one. |

| Supplementary Table S5. Risk of bias assessment of the included studies | | | | | |
| --- | --- | --- | --- | --- | --- |
| Study | D1 | D2 | D3 | D4 | Overall |
| Liang et al., 2025 | Unclear | Unclear | Low | Unclear | Unclear |
| Černý et al., 2025 | Low | Unclear | Low | Unclear | Unclear |
| Cao et al., 2025 | High | High | Unclear | Unclear | High |
| Pereira et al., 2025 | Unclear | Unclear | Unclear | Unclear | Unclear |
| Mendi et al., 2023 | Low | Unclear | Low | Unclear | Unclear |
| Wan et al., 2022 | Unclear | Unclear | Low | Unclear | Unclear |
| Wang et al., 2021 | High | High | Unclear | Unclear | High |
| Cuocolo et al., 2020 | Low | Unclear | Low | Unclear | Unclear |
| Zeynalova et al., 2019 | Unclear | Unclear | Low | Unclear | Unclear |

**
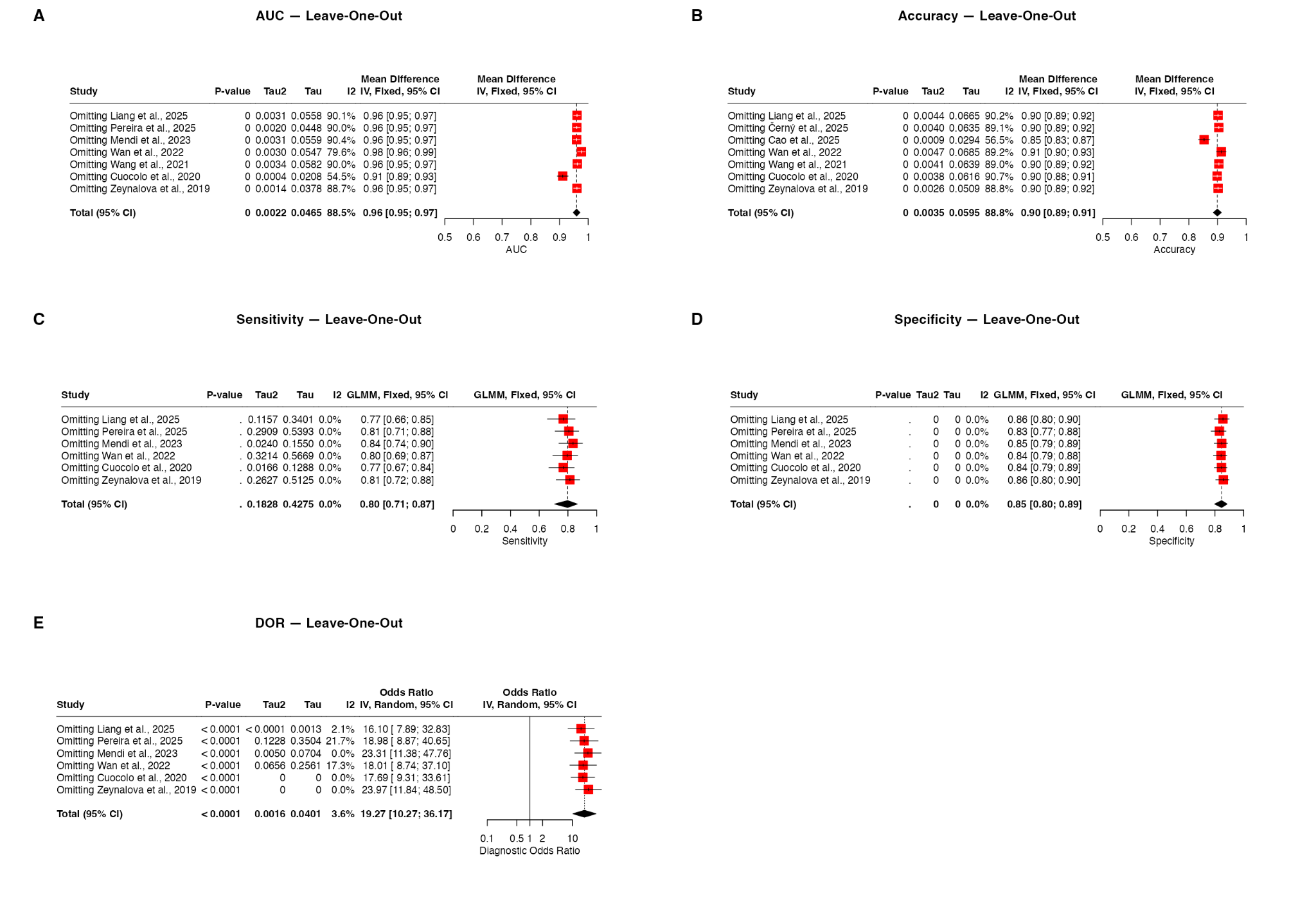
**

**Supplementary Figure S1.** Leave-one-out influence analysis for pooled diagnostic performance of machine learning models predicting pituitary adenoma consistency. Panels: (A) Area under the curve (AUC), (B) Accuracy, (C) Sensitivity, (D) Specificity, and (E)
